# Evaluating processing methods of hybrid rye fed to growing beef steers

**DOI:** 10.1093/tas/txag133

**Published:** 2026-08-31

**Authors:** Colin T Tobin, Zachary K Smith, Karl F Hoppe, Zachary E Carlson, Warren C Rusche

**Affiliations:** Department of Agronomy, Kansas State University, Manhattan, KS 66506, United States; Department of Animal Science, South Dakota State University, Brookings, SD 57007, United States; Carrington Research Extension Center, North Dakota State University, Carrington, ND 58421, United States; Department of Animal Sciences, North Dakota State University, Fargo, ND 58108, United States; Department of Animal Science, South Dakota State University, Brookings, SD 57007, United States

**Keywords:** feedlot, hybrid rye, steers, backgrounding

## Abstract

Crossbred *Bos taurus* beef steers (*n* = 150; initial shrunk body weight [BW] = 260 ± 22.2 kg) were used in a 63-d feedlot experiment to evaluate the effects of rolled (RRYE) and ground (GRYE) hybrid rye as replacements for dry-rolled corn (CON) on growth performance and comparative net energy (NE) values in diets fed to backgrounding steers. Rye from a single hybrid (KWS Bono) was processed using either a roller mill or a hammermill to achieve geometric mean particle diameters of 1780 µm and 731 µm, respectively. Three treatments were used in a randomized complete block design (*n* = 5 pens/treatment; 10 steers/pen), in which dry-rolled corn was completely replaced by RRYE or GRYE. Performance-adjusted NE was calculated using average daily gain (ADG), dry matter intake (DMI), and mean equivalent shrunk BW, and comparative NE values for rye were calculated using the replacement technique. Data were analyzed using the MIXED procedure of SAS 9.4 (SAS Inst. Inc., Cary, NC), with pen as the experimental unit. Treatment effects were tested using preplanned contrasts of CON vs. RYE and RRYE vs. GRYE. No differences between CON and RYE were detected (*P* ≥ 0.10) in ending BW, BW gain, ADG, DMI, gain:feed (G:F), or performance-adjusted NE for maintenance or gain (NEm, NEg). Steers fed GRYE tended to have greater BW gain (*P* ≤ 0.07) and ADG (*P* ≤ 0.06), with no differences in ending BW, DMI, or G:F compared with those fed RRYE. The concentration of NEg was calculated assuming dry rolled corn has an NEg concentration (Mcal/kg) of 1.49. Estimated replacement NEm and NEg values for RRYE included at 22% of diet DM were 1.91 and 1.25 Mcal/kg, respectively, whereas NEm and NEg values for GRYE were 2.29 and 1.58 Mcal/kg, respectively. Hybrid rye had a similar NE value to corn when fed in a high-forage backgrounding diet.

## Teaser

Hybrid rye performed similarly to corn in growing cattle diets and supported normal growth and feed efficiency. Grinding rye may further improve cattle performance, highlighting hybrid rye as a promising alternative feed grain for beef producers.

## Introduction

In the Midwest and Northern Great Plains, corn (*Zea mays* L.) and soybeans (*Glycine max* L.) are the typical cash crops grown as either monoculture or two-crop rotation (Rorick and Kladivko 2017). Recently, methods to enhance sustainability and resilience by improving soil properties, including changes in management (ie no-till vs conventional tillage; (Alhameid et al. 2017, 2019), increasing crop diversity (ie cover crops; (Rorick and Kladivko 2017), and grazing (Tobin et al. 2020; Bansal et al. 2022) have been shown to improve soil health parameters. The use of alternative crops, such as winter small grain annuals, can provide weed suppression (Hodgskiss et al. 2020), improve water infiltration rate, and cycle nitrogen (Otte et al. 2019). Cereal rye (*Secale cereale* L.) offers flexibility to producers when included in crop rotations. Rye can be grazed, hayed, or ensiled for forage production, or harvested as grain and straw (Rusche et al. 2020). Because rye is typically harvested for grain in early- to mid-summer, rye can readily be utilized for fall weaning and backgrounding while producers await autumn row crop harvest. However, integrating rye into crop production systems has been slow in U.S. cropping systems (Sadeghpour et al. 2021).

Cereal grains, including rye, are susceptible to ergot during flowering when exposed to extended cold and wet periods. Created as a secondary metabolite of the genus *Claviceps*, ergot alkaloids (EA) are toxic to animals if consumed in sufficient amounts (Coufal-Majewski et al. 2016). Rye grain use in feedlot cattle diets has been limited due to risks of ergotism. Recently, germplasm hybridization for improved yields and reduced ergot accumulation has been a goal of rye breeders.

Previous research has shown the applicability of hybrid rye to partially replace both dry rolled corn (DRC; Rusche et al. 2020; Buckhaus et al. 2021; Podversich et al. 2025b) and barley grain (Zhang et al. 2024a, 2024b) in finishing rations when concentrate inclusions exceeded 60% of dietary dry matter (DM). Complete replacement of DRC with hybrid rye did reduce average daily gain (ADG) and feed efficiency resulting in lesser net energy values for both unprocessed hybrid rye (NEm and NEg value of 1.73 and 1.11 Mcal/kg; Buckhaus et al. 2021) and rolled rye (NEm and NEg value of 1.91 and 1.26 Mcal/kg; Rusche et al. 2020) when compared to DRC (NEm and NEg values of 2.17 and 1.49 Mcal/kg; NASEM 2016). However, the net energy for maintenance (NEm) and net energy for gain (NEg) values of ground hybrid rye have not been well characterized. Previous research has also demonstrated that hybrid rye possesses greater starch degradability and altered ruminal fermentation characteristics compared with traditional cereal grains, potentially increasing susceptibility to rapid ruminal fermentation (Zhang et al. 2024a; 2024b).

Backgrounding rations often aim for a controlled rate of gain to increase frame size through increased muscle synthesis and skeletal development while limiting fat deposition (Perillat et al. 2004). Hybrid rye may serve as an alternative to corn grain because of its greater CP concentration (11.19% DM) while still providing supplemental energy to backgrounding rations. However, utilization of uncleaned hybrid rye in backgrounding diets remains limited due to its novelty and concerns regarding EA toxicity. Therefore, the objectives of this experiment were to (1) evaluate the effects of replacing DRC with hybrid rye on dry matter intake (DMI), ADG, gain efficiency, and estimate dietary net energy values in backgrounding steers, and (2) determine whether hybrid rye processing method (rolled vs ground) influenced DMI, ADG, and gain efficiency. We hypothesized that steers fed hybrid rye would have similar growth performance to steers fed DRC and that steers fed ground hybrid rye would exhibit improved growth performance compared with steers fed rolled hybrid rye.

## Materials and methods

All procedures involving the use of animals in this experiment were approved by the North Dakota State University Institutional Animal Care and Use Committee (approval number IACUC20220074). The experiment was conducted at the North Dakota State University Carrington Research Extension Center (CREC) located near Carrington, ND.

### Experimental design and treatments

Three treatments were used in a randomized complete block design to evaluate animal performance traits during the backgrounding phase. Hybrid rye was completely substituted on DM basis for DRC in the backgrounding ration as follows: a basal diet formulated with 22% corn grain (control diet; CON) on a DM basis with two additional diets formulated with either ground (GRYE) or rolled (RRYE) hybrid rye (Table 1). Diets were not reformulated to equalize energy or crude protein concentrations among treatments because the primary objective was to evaluate hybrid rye as a direct replacement for DRC under producer-relevant feeding conditions. The control diet was formulated to meet nutrient requirements for steers to achieve an ADG of 1.0 kg.

**Table 1:** Composition of experimental backgrounding diet treatments, day 1 to day 62 (DM basis).

Item	Treatment**^a^**		
	CON	RRYE	GRYE
**Ingredient Composition, % DM basis**			
**DRC**	22.3	0	0
**Hybrid Rye**	0	22.4	22.3
**MDGS^b^**	17.4	17.4	17.4
**Corn Silage**	34.2	34.2	34.3
**Barley Hay**	23.4	23.4	23.4
**Dry Supplement^c^^,^^d^**	1.8	1.7	1.7
**Limestone^d^**	0.9	0.9	0.9
**Nutrient Composition^e^**			
**NEm, Mcal/kg**	1.69	1.68	1.68
**NEg, Mcal/kg**	1.08	1.06	1.06
**CP, %**	12.72	13.31	13.25
**NDF, %**	40.20	41.06	41.13
**ADF, %**	24.93	23.41	23.46
**Ash, %**	6.33	6.59	6.49
**EE, %**	3.10	2.75	2.77
**Starch Content**			
**Grain, %**	67.76	58.22	
**Ration, %**	26.97	24.90	24.88

a From d 1 to 62, pens were fed either a forage-based diet with dry rolled corn (DRC[CON]), dry rolled rye (RRYE), or ground hybrid rye (GRYE) as the concentrate.

b Modified distiller grains with solubles.

c Provided 30 g/ton of monensin, as well and minerals to exceed requirements (NASEM 2016).

d Fed at fixed rate per animal.

e Dry matter was measured weekly, nutrient composition were analyzed from composite ingredient samples. Net energy values were estimated from NASEM (2016). Net energy for maintenance (NEm), Net energy for gain (NEg), Crude protein (CP), neutral detergent fiber (NDF), Acid detergent fiber (ADF), ether extract (EE).

### Animals, initial processing, study initiation

One hundred fifty cross-bred steers (initial shrunk body weight [BW] = 260 ± 22.2 kg) were used in this experiment. Steers were consigned to the Dakota Feeder Calf Show (DFCS) from multiple ranches and processed at the Turtle Lake Weigh Station, ND, vaccinated against infectious bovine rhinotracheitis, bovine viral diarrhea types 1 and 2, parainfluenza-3 virus, *Mannheimia haemolytica* and bovine respiratory syncytial virus (Pyramid 5 + Presponse SQ; Boehringer Ingelheim Animal Health, Duluth, GA; Inforce 3, Zoetis, Parsippany, NJ), clostridial species (Bar-Vac 7/Somnus; Boehringer Ingelheim), administered pour-on moxidectin (Cydectin, Bayer, Shawnee Mission, KS) and an oxytetracycline injection (Noromycin 300 LA, Norbrook Inc., Lenexa, KS). Additionally, steers were administered a steroidal implant (200 mg progesterone and 20 mg estradiol benzoate; Synovex S, Zoetis). Following processing, steers were delivered to the CREC on 15 October 2022. The experiment was initiated on 24 October 2022, after steers were adapted to the CON diet.

Body weights were collected on 2 consecutive days, with the mean weight used as the initial body weight. Steers were stratified by weight and randomly assigned to pens. Steers were fed in eight dirt and seven cement-surfaced pens (*n* = 15; 10 steers/pen), resulting in five replications per treatment with 50 steers per treatment. Calves were housed in pens which had 1 m of concrete bunk space, 3.5 m of concrete feed apron, and 39.1 m^2^ of pen space per steer.

### Diets and intake management

Steers were fed once daily at 0800 h. Bunks were managed using a slick bunk management program so that bunks were devoid of feed at approximately 0700 h. Ingredient samples were collected weekly and DM calculated after drying in a forced-air oven at 60°C until no changes in weight were recorded. Ration adjustments were made from weekly DM values for each ingredient. Three steers (2 from CON and 1 from RRYE) died during the study, which included 2 from pneumonia and 1 from an accident in the pen. Steers that died or were removed from the study were assumed to have consumed feed equal to the pen mean DMI up to the point of removal or death. All data are reported on a deads-in basis.

All hybrid rye was received from a single source and was the same hybrid (KWS Bono, KWS Cereals, LLC; Champaign, IL). Hybrid rye was processed through rolling or grinding. Rolled hybrid rye was processed by passing through Roskamp model J roller mill (Lone Star Enterprises, Lennox, SD). The rolls were 61 × 22 with 3.1 corrugations per centimeter in a round bottom v pattern and ran at 850 rpm at a 1:1 ratio. Corrugations in one roller were straight, while the second roller was machined with a 12.7 cm spiral design. Ground hybrid rye was processed by passing through a hammermill using a 0.64 cm screen offsite at a local feedstore. The hybrid rye delivered to CREC was sampled and analyzed for EA. Ergot alkaloid concentrations were analyzed at the NDSU Veterinary Diagnostic Laboratory (Fargo, ND; Table 2). Total ergot alkaloid concentration of the hybrid rye grain was 308 µg/kg (0.308 mg/kg; Table 2), which was below the recommended limit of 2 mg/kg in cattle diets to minimize negative effects on health and performance (Coufal-Majewski et al. 2016; Friskop et al. 2018).

**Table 2:** Hybrid rye ergot alkaloid concentration.

Ergot Alkaloid	Concentration, µg/kg
**Ergosine**	22
**Ergotamine**	< 20
**Ergocornine**	64
**Ergocryptine**	85
**Ergocristine**	< 20
**Ergosinine**	<20
**Ergotaminine**	<20
**Ergocorninine**	<20
**Ergocryptinine**	< 20
**Ergocristinine**	< 20
**Total**	308

All corn was received from a single local source. Corn was processed by passing through Roskamp model J roller mill (Lone Star Enterprises, Lennox, SD). The rolls were 61 × 22 with 3.1 corrugations per centimeter in a round bottom v pattern and ran at 850 rpm at a 1:1 ratio. Corrugations in one roller were straight, while the second roller was machined with a 12.7 cm spiral design.

Processed hybrid rye and dry rolled corn samples were analyzed for particle size distribution and geometric mean diameter at the NDSU Northern Crops Institute in Fargo, ND (Table 3). Samples were split and a 100-g subsample was weighed and sieved through a set of 13 sieves (3350 μm, 2360 μm, 1700 μm, 1180 μm, 850 μm, 600 μm, 425 μm, 300 μm, 212 μm, 150 μm, 75 μm, 53 μm, and pan) using a sieve shaker for 10 min. After the sample was shaken, the weight of the material on each sieve was recorded. No dispersion agents were used in the analysis.

**Table 3:** Dry rolled corn, ground rye, and rolled rye particle size distribution, geometric mean diameter (GMD), and geometric mean diameter SD (GMDSD).

Item	Dry Rolled Corn (%, retained)	Ground Rye (%, retained)	Rolled Rye (%, retained)
**Screen size, µm**			
**3350**	68.1%	0.0%	0.0%
**2360**	15.0%	0.4%	15.8%
**1700**	8.1%	13.4%	57.0%
**1180**	4.2%	26.8%	19.8%
**850**	1.6%	23.0%	4.4%
**600**	1.1%	11.6%	1.7%
**425**	0.7%	4.2%	0.4%
**300**	0.4%	3.8%	0.0%
**212**	0.2%	4.2%	0.3%
**150**	0.2%	1.5%	0.4%
**106**	0.2%	1.7%	0.0%
**75**	0.0%	1.9%	0.2%
**53**	0.1%	0.7%	0.0%
**Pan**	0.0%	6.8%	0.0%
**GMD, µm**	3624.36	731.09	1780.29
**GMDSD**	1.78	2.854	2.741

Wet chemistry analyses of individual ingredients were conducted at Dairyland Laboratories, Inc. (Arcadia, WI) prior to study initiation for use in initial diet formulation. Weekly ingredient samples were composited on a DM basis and analyzed to determine diet composition. Analyses included ash (method 942.05; AOAC 1996), ether extract (EE; method 920.39; AOAC 1996), neutral detergent fiber (NDF; 2002.04; AOAC, 2005), acid detergent fiber (ADF; method 973.18; AOAC 1996), and crude protein (CP; method 990.03; AOAC 1996). Starch concentrations of corn silage, corn, and hybrid rye were also analyzed (Hall et al. 2015) and total dietary starch concentration was calculated for each ration (Table 1).

### Cattle management and data collection

Steer BW were recorded at the time of study initiation (day 0), and days 1, 2, 28, 62, and the morning of study termination on day 63. Body weights were measured before the morning feeding with a 4% pencil shrink applied for all weights.

Daily energy gain (EG; Mcal/day) was used to calculate performance adjusted NE (paNE): EG = [ADG from d 1 to 63]^1.097^ × 0.0557 W^0.75^, where W is the mean equivalent shrunk BW [shrunk BW × (478/AFBW), kg; NRC 1996] for the period from day 1 to day 63. Maintenance energy required (EM; Mcal/d) was calculated by the following equation: EM = 0.077BW^0.75^ (Lofgreen and Garrett 1968), where BW is the mean shrunk BW (using the average of finished carcass-adjusted ending BW and BW from day 1). Using the estimates required for maintenance and gain, the paNE for maintenance (paNEm) and gain (paNEg) values (Owens and Hicks 2019) of the diet were generated using the quadratic formula: $x=\frac{-b\pm \sqrt{b^{2}}-4ac}{2c}$, where x = NEm, Mcal/kg, a = −0.41 EM, b = 0.877 EM + 0.41 DMI + EG, c = −0.877 DMI, and NEg was determined from: 0877 NEm −0.41 (Zinn and Shen 1998; Zinn et al. 2008).

Comparative NEm values for hybrid rye were estimated using the replacement technique. Given that the NEm concentration of DRC is approximately 2.17 Mcal/kg (NASEM 2016), the comparative NEm values for both hybrid rye processing methods were estimated as follows (Estrada-Angulo et al. 2019; Rusche et al. 2020): Hybrid rye NEm (Mcal/kg = [(test diet paNEm–control diet paNEm)/RYE] + 2.17), where RYE represents the inclusion of rye (0.221) that replaced DRC in the diet. The concentration of NEg was calculated similarly assuming DRC has NEg concentration (Mcal/kg) of 1.49 (NASEM 2016).

### Statistical analysis

Data were analyzed as a randomized complete block design using the MIXED procedure of SAS 9.4 (SAS Inst Inc., Cary, NC; Littell et al. 2006) with pen serving as the experimental unit. The model included the fixed effect of dietary treatment (CON, RRYE, GRYE) and weight block (1–5). Least squares means were generated using the LSMEANS statement of SAS and treatment effects were evaluated using orthogonal polynomials (Steel and Torrie 1960). Pre-planned contrasts (CON vs RYE; RRYE vs GRYE) were analyzed. An *α* of 0.05 or less determined significance, and tendencies are discussed between 0.05 and 0.10.

## Results

Ending BW was not influenced (*P* = 0.27) by dietary treatment (Table 4). At the end of the 63-d study, steers assigned to CON, RRYE, and GRYE had an ending BW of 350, 345, and 353 kg, respectively. Additionally, total BW gain was not influenced (*P* = 0.79) by dietary treatment. Overall, steers assigned to CON, RRYE, and GRYE gained 93, 89, and 93 kg, respectively. Steers assigned GRYE tended to have 8.2% increased BW gain compared to those assigned to RRYE (*P* = 0.07). No weight gain differences were detected in the contrasts between CON vs RYE (*P* = 0.91).

**Table 4:** Influence of replacing dry rolled corn with hybrid rye on interim period steer growth performance.

	Dietary treatment^a^				*P*-value^b^	
	CON	RRYE	GRYE	SEM	CON vs Rye	GRYE vs RRYE
**Initial BW, kg^c^**	261	259	260	2.1	0.32	0.92
**Initial to day 28**						
**BW day 28, kg^c^**	323	317	319	2.2	0.07	0.50
**ADG, kg**	1.74	1.60	1.64	0.056	0.11	0.63
**DMI, kg**	7.03	6.90	6.83	0.133	0.33	0.70
**G:F**	0.25	0.23	0.24	0.007	0.15	0.54
**Day 29–64**						
**BW day 64, kg^c^**	365	359	367	3.4	0.76	0.12
**ADG, kg**	1.16	1.19	1.36	0.060	0.16	0.08
**DMI, kg**	8.90	9.20	9.37	0.216	0.18	0.60
**G:F**	0.13	0.13	0.15	0.007	0.46	0.21
**Day 1–64**						
**Ending BW, kg^c^**	365	359	367	3.4	0.76	0.12
**BW gain, kg**	93	89	97	2.7	0.91	0.07
**ADG, kg**	1.47	1.42	1.54	0.042	0.89	0.06
**DMI, kg**	8.07	8.18	8.24	0.155	0.49	0.81
**G:F**	0.18	0.17	0.19	0.007	0.98	0.13
**Predicted dietary net energy based on performance**						
**paNE, Mcal/kg^d^**						
**Maintenance**	1.81	1.75	1.84	0.037	0.77	0.61
**Gain**	1.18	1.13	1.20	0.033	0.77	0.61
**Observed/expected dietary NE^e^**						
**Maintenance**	1.07	1.04	1.09	0.023	0.94	0.49
**Gain**	1.09	1.06	1.13	0.312	0.82	0.35
**Estimated NE Value of Hybrid Rye processing, Mcal/kg^f^**						
**Maintenance**	–	1.91	2.29	–	–	–
**Gain**	–	1.25	1.58	–	–	–

a From d 1 to 62, pens were fed either a forage-based diet with dry rolled corn (CON), dry rolled rye (RRYE), or ground hybrid rye (GRYE) as the concentrate.

b CON vs. Rye = CON vs. RRYE, GRYE.

c BW was shrunk 4% to account for digestive tract fill.

d Owens and Hicks (2019).

e paNE/tabular trial NE (from Preston 2017).

f Net energy values for Rye derived using the replacement technique, assuming that NEm and NEg of DRC are 2.17 and 1.49 Mcal/kg, respectively (NASEM 2016).

No differences in ADG due to dietary treatment were detected (*P* = 0.16; Table 4) throughout the study. For the entire 63-d study, steers assigned to CON, RRYE, and GRYE had an ADG of 1.47, 1.42, and 1.54 kg, respectively. No differences in ADG were detected during the first 28 d (*P* = 0.23) but there was a tendency for the GRYE dietary treatment to have increased ADG compared to the RRYE dietary treatment from d 28–63 (*P* = 0.08). No differences were detected in preplanned contrasts during day 1–28 (*P* > 0.10). Steers assigned to RRYE tended to have lower ADG throughout the study than those assigned GRYE (*P* = 0.06).

No differences in DMI due to dietary treatment were detected (*P* = 0.75) throughout the study (Table 4). Across the 63-d study, steers assigned to CON, RRYE, and GRYE had an average daily dry matter intake of 7.07 8.18, 8.24 kg of feed, respectively. Throughout the study, no differences between contrasts were detected (*P* > 0.10).

No differences in G:F due to dietary treatment were detected (*P* = 0.28; Table 4). Across the 63-d study, steers assigned to CON, RRYE, and GRYE gained 0.18, 0.17, and 0.19 kg per kg of feed consumed, respectively.

No differences in paNE due to dietary treatment were detected (*P* = 0.33). Additionally, no differences between the pre-planned contrasts were detected (*P* ≥ 0.15). Similarly, no differences in observed/expected dietary NE were detected (*P* = 0.32) or among the preplanned contrasts (*P* ≥ 0.15). Lastly, the estimated NEm and NEg value were 1.91 and 1.25 Mcal/kg and 2.29 and 1.58 Mcal/kg for RRYE and GRYE, respectively.

## Discussion

The estimated dietary NE values and growth performance for hybrid rye observed in the present study support its use as a substitute for DRC in backgrounding diets. Estimated NEm and NEg values for hybrid rye when included at 22.1% of dietary DM were 2.00 and 1.34 Mcal/kg for rolled rye and 2.09 and 1.42 Mcal/kg for ground rye, respectively. These values were numerically greater than rye tabular values (1.97 and 1.32 Mcal/kg; NASEM 2016; 1.90 and 1.23 Mcal/kg; Preston 2017), and greater than values for rolled rye (1.90 and 1.25 Mcal/kg; Rusche et al. 2020) and unprocessed, whole rye (1.73 and 1.11 Mcal/kg; Buckhaus et al. 2021).

Processing hybrid rye through a hammermill produced substantially smaller particles than rolling and was associated with greater apparent NEm and NEg values. Although the barriers to starch digestion differ between rye and corn, increased processing intensity may have improved energy utilization of hybrid rye in the present study. This enhanced starch availability likely contributed to the numerically greater growth performance observed compared to those fed rolled hybrid rye. In diets where rye grain was fed to finishing steers and was processed to different particle sizes, increased particle size was associated with reduced organic matter digestibility and a tendency for reduced starch digestibility (Podversich et al. 2025a). In that study, finishing cattle fed hammer-milled rye gained similarly to steers fed rolled rye but consumed more DM, resulting in lesser NE values (Podversich et al. 2025a) in contrast to the current experiment where GRYE tended to increase ADG and G:F with no effect on DMI. The hammer-milled rye used in the finishing cattle experiment was less extensively processed compared to the GYRE in the current experiment (1901 µm vs. 731 µm, respectively). In contrast, Zinn et al. (2011) noted minimal differences in NE values comparing ground maize with coarse processing (rolling/cracking).

Studies evaluating hybrid rye in high-concentrate, finishing diets have reported reduced ADG and feed efficiencies when rye replaces corn at high inclusion rates (Rusche et al. 2020; Buckhaus et al. 2021; Podversich et al. 2025b). In such systems, the lower starch content of rye relative to corn may reduce dietary NE supply under ruminal conditions characterized by rapid starch fermentation and faster passage rates. In contrast, the backgrounding diets used in the present study contained approximately 60% forage, which may increase ruminal retention time and allow greater opportunity for digestion of the hybrid rye. Additionally, the absence of a DMI response may be related to the lower grain and greater forage inclusion compared with high-concentrate finishing diets. Rye grain is characteristically more rapidly fermentable compared to other cereal grains (Krieg et al. 2017; Ratjar et al. 2020; Rusche and Smith 2024; Zhang et al. 2024b). In a backgrounding diet with greater roughage content, feeding a more rapidly fermentable starch source may have less potential to induce sub-acute acidosis compared to finishing diets with less roughage, which could explain why more extensively processed rye grain tended to increase gain and efficiency in the current experiment. Furthermore, because grain represented a smaller proportion of the total dietary dry matter, any inherent energetic differences between rye and corn would be less detectable. These factors may explain why hybrid rye performed comparably to corn in the present study and why processing intensity exerted a more pronounced influence on animal performance than grain source alone.

Total ergot alkaloid concentration of the hybrid rye used in this study was 0.308 mg/kg, which was well below the recommended threshold of 2 mg/kg for cattle diets (Coufal-Majewski et al. 2016; Friskop et al. 2018). Ergot alkaloid consumption has been associated with reduced feed intake, impaired thermoregulation, and decreased animal performance when concentrations exceed recommended limits. Despite complete replacement of DRC with hybrid rye, DMI and ADG were not affected, suggesting that the ergot alkaloid concentration of the grain used in this study was insufficient to negatively influence animal performance.

## Conclusion

In conclusion, replacement of DRC with processed hybrid rye at 22.1% of diet DM did not affect DMI, ADG, feed efficiency, or estimated dietary net energy values in backgrounding steers. Ground hybrid rye tended to increase ADG and final BW compared with rolled hyrbrid rye and resulted in numerically greater estimated NEm and NEg value, suggesting the processing intensity my influence energy utilization when hybrid rye is incorporated into forage based backgrounding diets. The absence of intake depression or health-related concerns in this study also supports the safe use of modern low-ergot hybrid rye cultivars in backgrounding systems when ergot alkaloid concentrations are maintained below recommended thresholds.

## List of abbreviations

ADF: Acid detergent fiber
ADG: Average daily gain
AFBW: Adjusted final body weight
BW: Body weight
CON: Control treatment
CP: Crude protein
CREC: Carrington Research Extension Center
DFCS: Dakota Feeder Calf Show
DM: Dry matter
DMI: Dry matter intake
DRC: Dry rolled corn
EA: Ergot alkaloids
EE: Ether extract
EG: Daily energy gain
EM: Maintenance energy required
G:F: Gain to feed ratio
GRYE: Ground rye grain
NDF: Neutral detergent fiber
NE: Net energy
NEg: Net energy for gain
NEm: Net energy for maintenance
paNEg: Performance adjusted net energy for gain
paNEm: Performance adjusted net energy for maintenance
RRYE: Rolled rye grain
W: Mean equivalent shrunk body weight
